# Presence of interplate channel layer controls of slip during and after the 2011 Tohoku-Oki earthquake through the frictional characteristics

**DOI:** 10.1038/s41598-021-86020-9

**Published:** 2021-03-19

**Authors:** Ryoko Nakata, Takane Hori, Seiichi Miura, Ryota Hino

**Affiliations:** 1 Graduate School of Science, Tohoku University, 6-6, Aramaki-aza-aoba, Aoba-ku, Sendai, 980-8578 Japan; 2 Research and Development Center for Earthquake and Tsunami Forecasting (FEAT), Research Institute for Marine Geodynamics (IMG), Japan Agency for Marine-Earth Science and Technology (JAMSTEC), 3173-25 Showa-machi, Kanazawa-ku, Yokohama, 236-0001 Japan; 3 Subduction Dynamics Research Center (SDR), Research Institute for Marine Geodynamics (IMG), Japan Agency for Marine-Earth Science and Technology (JAMSTEC), 3173-25 Showa-machi, Kanazawa-ku, Yokohama, 236-0001 Japan

**Keywords:** Geophysics, Seismology, Tectonics

## Abstract

There are significant differences between the middle and southern segments of the Japan Trench in terms of the seismic and aseismic slips on the plate interface and seismic velocity structures. Although the large coseismic slip of the 2011 Tohoku-Oki earthquake was limited to the middle segment, the observed negative residual gravity anomaly area in the southern segment corresponds to the postseismic slip area of the Tohoku-Oki earthquake. A density distribution model can explain the different slip behaviours of the two segments by considering their structural differences. The model indicates that the plate interface in the south was covered with a thick channel layer, as indicated by seismic survey imaging, and this layer resulted in a residual gravity anomaly. Numerical simulations which assumed evident frictional heterogeneity caused by the layer in the south efficiently reproduced M9 earthquakes recurring only in the middle, followed by evident postseismic slips in the south. This study proposes that although the layer makes the megathrust less compliant to seismic slip, it promotes aseismic slips following the growth of seismic slips on the fault in an adjacent region.

## Introduction

Many studies which investigated the spatiotemporal distributions of various seismic and geodetic events and underground structures have demonstrated that there are conspicuous differences between the middle and southern segments of the Japan Trench. The shallow area off the coast of Fukushima Prefecture, i.e. the southern segment of the trench, is known to have few large earthquakes. Interplate coupling in the southern segment is considerably weaker than that off the coast of Miyagi Prefecture, the middle segment of the trench^1^. Seismic and geodetic data, as well as marine geophysical and geological data, have demonstrated that the large coseismic slip in the shallow area which caused the 2011 Tohoku-Oki earthquake was limited to the middle segment^2–5^. Additionally, palaeoseismological evidence collected along the Japan Trench demonstrated that deep-sea turbidites which originated from large earthquakes potentially similar to the 2011 Tohoku-Oki earthquake are recorded only in the middle segment^6^. The characteristics of spatial heterogeneity along the trench may not have changed over an extended period.

**Figure 1:**
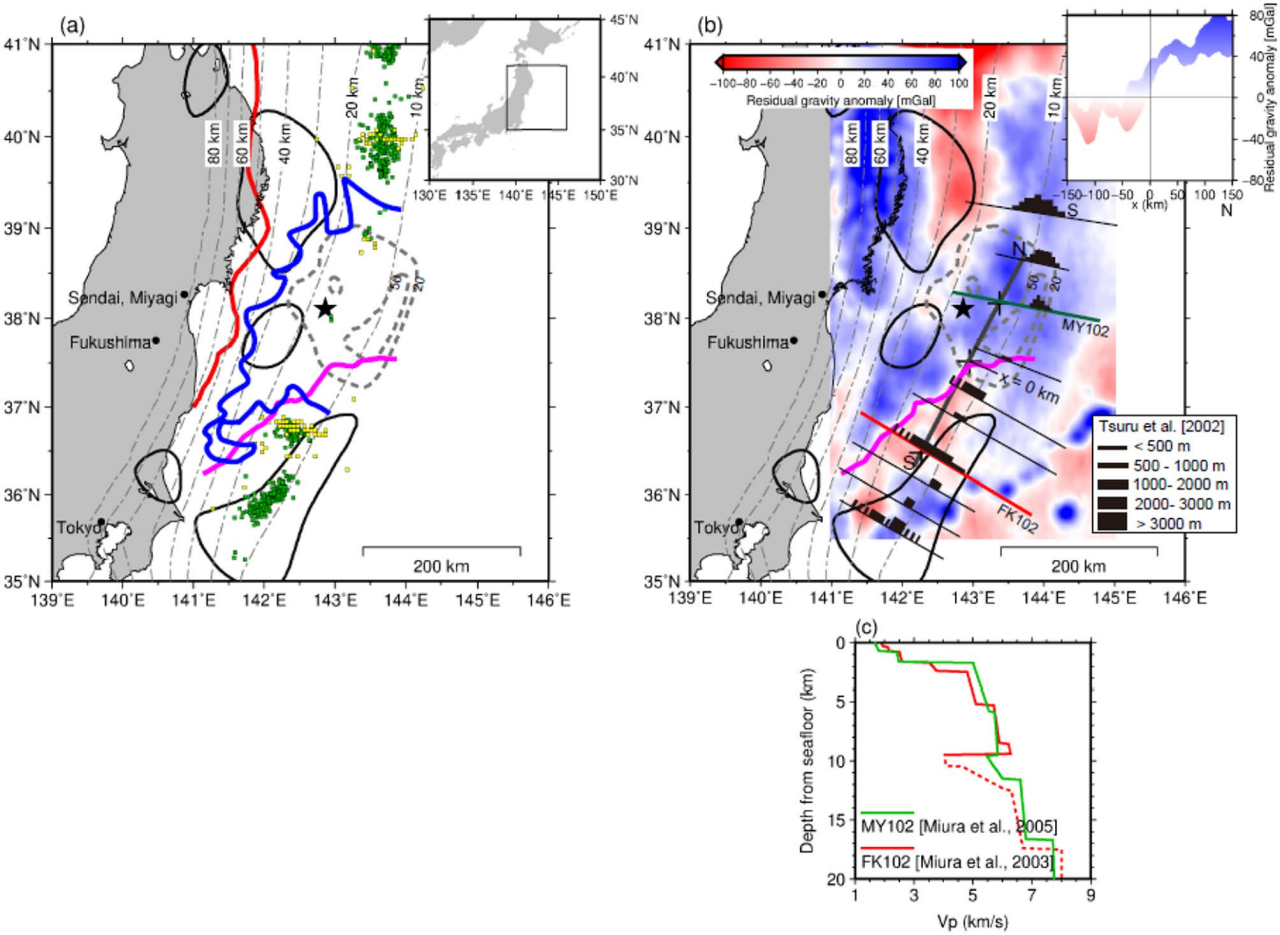
(**a**) Distributions of seismic/aseismic events along the Japan Trench. The black solid lines indicate the cumulative distribution of the estimated postseismic slip (> 0.4 m) for 0.6 years of the 2011 Tohoku-Oki earthquake^7^. The grey dashed contours represent the coseismic slip distribution (> 20 m, > 50 m) of the Tohoku-Oki earthquake^3^. Green squares indicate tremors^10^. Yellow squares denote very-low-frequency earthquakes^10^. Red and blue curves indicate the down-dip limit of the interplate earthquake distribution^53^ and the outer edge of the large-slip zone during the mainshock rupture^16^, respectively. The black star indicates the hypocentres of the Tohoku-Oki earthquake. The thin, black, dashed contours extending in the north–south direction indicate the depth (km) to the upper surface of the descending plate^41^. The magenta line is the forearc segment boundary^15^. The rectangle in the inset represents the location of the study area. (**b**) Colours indicate the residual gravity anomaly (modified from Bassett et al.^15^). The magenta line is the same as in (**a**). The black bars indicate the low-velocity sedimentary units observed on MCS sections^18^. Green and red lines with subscripts MY102 and FK102 indicate survey lines^18,31,32^. Crosses on these lines indicate locations at which velocity profiles are shown in (**c**). The inset shows the profile of the residual gravity anomaly along the thick black line S–N (along the contour of approximately 13 km depth). (**c**) Seismic velocity profiles. Green and red lines indicate the P-wave velocity (Vp) off Miyagi and Fukushima (crosses on MY102 and FL102 in b) estimated from marine surveys^31,32^. The vertical axis indicates the depth from the seafloor. The lateral axis indicates the Vp. The dashed lines indicate a lower velocity resolution. There is a low-velocity zone at a depth of 2–5.5 km (upper crust, lower sedimentary layer) and a depth > 10 km (channel layer) at the southern segment (green line). However, the Vp = 6 km/s layer is widely distributed at the ~ 5.5–10 km depth, where it is directly above the plate interface of the middle and southern segments of the Japan Trench.

In contrast, postseismic slips were predominant in the southern segment^7,8^. Slow earthquakes include low-frequency earthquakes, tectonic tremors, and very-low-frequency earthquakes, which are distributed complementary to the coseismic slip and overlapping the postseismic slip area of the Tohoku-Oki earthquake^9–11^ (Fig. 1a).

The postseismic slip during April–December 2011 was estimated to be < 1.2 m^7^, and the 1-year postseismic slip following the 2011 earthquake was estimated to be approximately 2.5 m^12^ at the shallow portion of the southern segment of the Japan Trench. As of December 2017, the seafloor displacement analyses demonstrated that postseismic slip continued in the southern segment, and the average slip rate between January 2015 and December 2017 was approximately half of the average slip rate between March 2011 and December 2014 off the coast of Fukushima Prefecture (at the FUKU station)^13^.

Researchers have been working to determine the factors that control these complementary spatial distributions of slips along the Japan Trench. Liu and Zhao^14^ showed a relationship between the fault slip behaviour in the Tohoku-Oki earthquake and the P-wave tomography of the Tohoku forearc. They proposed that structural heterogeneities in both the overriding plate and atop the subducting plate controlled the processes of nucleation and rupture in the Tohoku-Oki earthquake. Bassett et al.^15^ found that the large coseismic slip zone of the 2011 earthquake is delimited by the evident negative residual gravity anomaly zone in the south. Evident hollow interplate seismicity after the 2011 earthquake, which is another indicator of the coseismic slip area of the M9 earthquake^16^, and past large thrust earthquakes are located outside the negative anomaly zone, whereas the distributions of postseismic slip from the Tohoku-Oki earthquake and slow earthquakes correspond to the zone^15^.

Barbot^17^ focused on down-dip segmentation of rupture styles; partial ruptures at the deeper part and a giant, super-cycle rupture at the entire velocity-weakening interface. Barbot^17^ presented two-dimensional simulations, which suggested that the rupture styles at the middle segment of the Japan Trench are controlled by both structural and frictional heterogeneity. Therefore, the spatial distributions of slip along the trench may also be explained by structural and frictional heterogeneity.

In this study, we focussed on the along-strike distribution of the sedimentary units observed on multichannel seismic (MCS) sections at the Japan Trench^18^. The sedimentary units represent a narrow wedge shape in the middle segment but exhibit a thin and broad distribution along the plate interface, forming a low-velocity channel layer^18^ in the shallow part of the southern segment. The subduction channel is an approximately 100–1000 m thick shear zone, and it is typically defined as the sedimentary layer between the downgoing oceanic crust and the base of the upper plate^19^. The borders of these two different characteristics in sediment distribution correspond well to the southern border of the extensive coseismic slip zone, as well as to the boundary delimiting the negative residual gravity anomaly area (Fig. 1b).

The channel layer that is broadly distributed in the southern Japan Trench is considered to have been developed following a previously subducted seamount, creating a large stress shadow zone behind them^20,21^. Many seafloor indentations on the trench’s landward slope in the area^22,23^ indicate that many seamounts, such as those on the seafloor of the incoming plate, have previously been subducted.

The correlation between the existence of the channel layer and the aseismic postseismic slip, as discussed herein, can be explained in the context of the predominance of fault creeping in subduction zones with a rugged seafloor of incoming plates^24^. The numerical models of Sun et al.^21^ suggested that aseismic or slow slip is favoured in the stress shadow located at the updip side of the subducting seamount, where anomalously high sediment porosity is expected. Subduction plate boundaries are deformation zones with a finite width^19^. Vannucchi et al.^19^ reviewed various patterns of internal deformation that occurred within the subduction channel layers. Wang and Bilek^24^ proposed broadly distributed deformation as a possible mechanism responsible for the creeping nature along rugged subduction interfaces.

In this study, inspired by the existence of subducting seamounts, we consider the internal deformation of the subduction channel layer^19,24^ increases the critical slip distance on a macroscopic scale, similar to that within a fault gouge layer in laboratory experiments^25,26^. Therefore, the presence of the channel layer in the wake of subducting seamounts makes the rugged subduction interface aseismic. The critical slip distance, which is a type of frictional parameter in the rate- and state-dependent friction law^27^, is a parameter which varies with surface roughness. Laboratory measurements and observations indicate that the thickness of the fault gouge layer is positively correlated with the critical slip distance^25,26^. In the framework of the friction law, where critical slip distances are large, aseismic slips are more likely to occur^28,29^. Then, we hypothesise that the large value of the critical slip distance spreads on the plate interface in the southern segment. This hypothesis is consistent with the aforementioned observation as some types of aseismic slips are observed in the southern segment, where a thick channel layer exists. Notably, the scope of this study is to explain the control of the channel layer on the M9 earthquake on a large spatial scale. Small-scale failures (such as aftershocks, low-frequency earthquakes, tectonic tremors, and very-low-frequency earthquakes) on a creeping fault on the macroscopic scale are possible owing to the multiscale heterogeneity^30^ of natural faults.

Based on these previous studies, the presence of the channel layer can be expressed as a large critical slip distance in terms of frictional mechanics. Considering that this theory is correct, then there was no massive coseismic slip during the mainshock of the 2011 event, whereas evident aseismic postseismic slip occurred in the area of negative residual gravity anomaly, reflecting the presence of a channel layer.

In this study, we semi-quantitatively confirmed that the presence of the channel layer is consistent with the observed negative residual gravity anomaly by constructing a crustal density model which is based on seismic velocity models. We also conducted three-dimensional numerical simulations of earthquake generation cycles along the Japan Trench by considering the presence of a channel layer with a characteristic slip distance value. By attempting to reproduce megathrust earthquake cycles along the trench with four frictional models, we revealed the control of structural heterogeneity on the occurrence of earthquakes and complementary spatial distributions of slip in the study region.

## Structural model expressing observed differences between the middle and southern segments

Bassett et al.^15^ explained that the evident difference in residual gravity anomaly between the middle segment, where large coseismic slip occurred, and the southern segment, which is characterised by shallow postseismic slip, could be explained by the large density contrast of the hanging wall side of the plate interface. They assumed a single-layered structure for the hanging wall and showed that the density contrast of ~ 180 kg/m^3^ in the layer accounts for the contrast in the observed residual gravity anomaly across the segment boundary.

However, the results of wide-angle active seismic experiments do not support structural differences throughout the hanging wall crust. According to the P-wave velocity (Vp) models reported by Miura et al.^31,32^ obtained for the middle and southern segments, in the deeper part of the crustal layer underlain by the plate boundary, the difference in Vp is insignificant (Fig. 1c) compared to the large density contrast to explain the residual gravity anomaly. According to the standard relationship between Vp and the density of the crustal materials^33^, a density contrast of 180 kg/m^3^ is equivalent to a Vp anomaly of approximately − 10%, which is not consistent with the Vp models using active seismic explorations (Fig. 1c).

**Figure 2:**
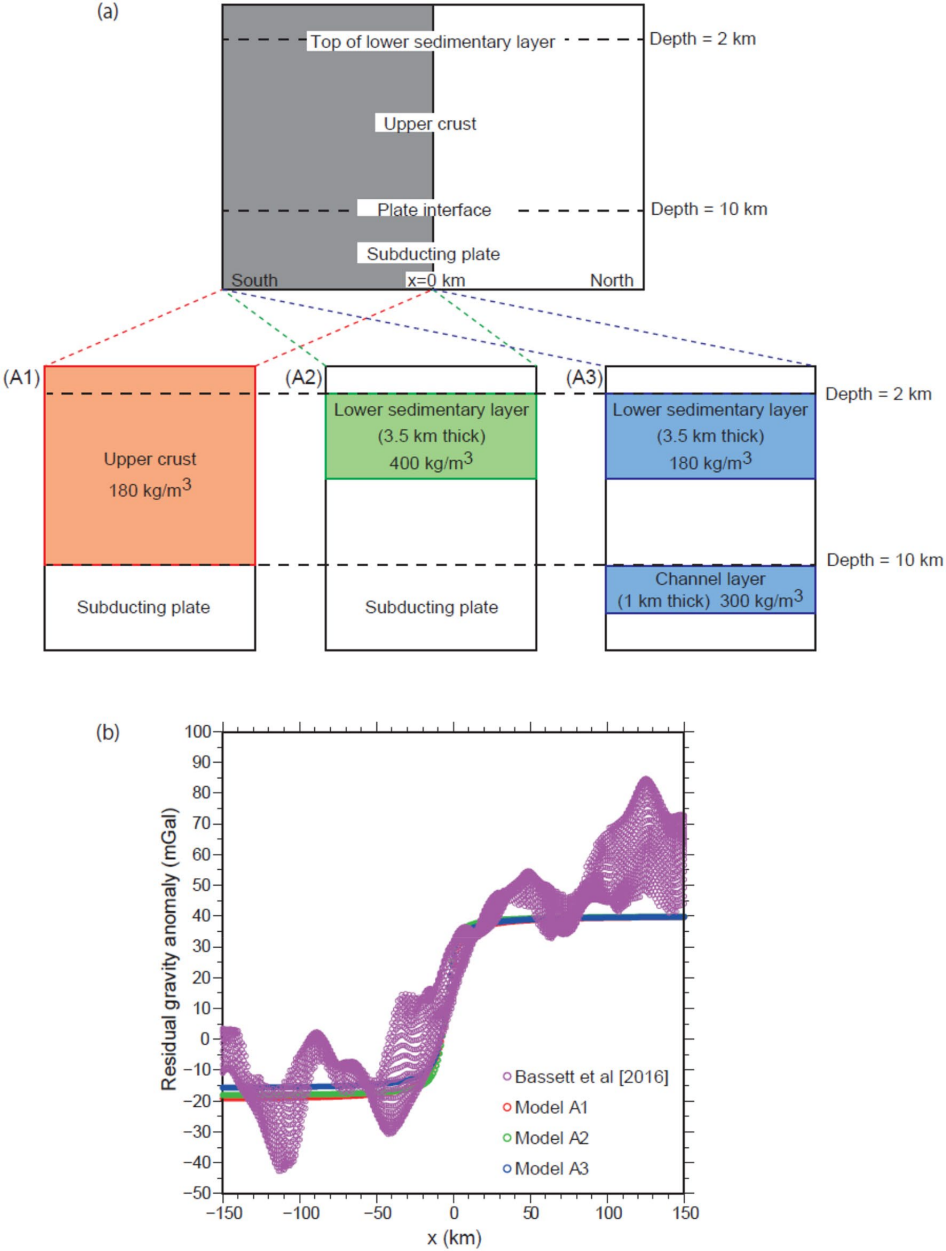
(**a**) Schematic of the density distribution model. Models A1–A3 represent the southern segment of the Japan Trench (grey area in the upper panel). (**b**) Residual gravity anomaly estimated from various density distribution models. Purple lines indicate the residual gravity anomaly (from Bassett et al.^15^). Red, green, and blue lines indicate results obtained using Models A1, A2, and A3, respectively.

Herein, we hypothesise that the presence of the channel layer imaged by seismic surveys in the southern segment accounts for the observed spatial pattern of the residual gravity anomaly. We confirmed this hypothesis by performing a simple gravity calculation with a horizontally stratified two-dimensional (2D) model composed of two segments, which correspond to the middle and southern segments of the Japan Trench forearc, respectively (Method; Fig. 2a).

We have presented three density models which accurately explain the observed residual gravity anomaly equally (Fig. 2). One is composed of a single crustal layer (Model A1) similar to that presented by Basset et al.^15^. The other two are based on low-Vp zones identified by Miura et al.^31,32^. There are two significant low-Vp zones, which are the candidates for the layers of a negative density anomaly, accounting for the negative residual gravity anomaly at depths which range from 2 to 5.5 km and 10 to 11 km (Fig. 1c). The model that assumes a shallower layer with a thickness of 3.5 km, which is solely responsible for the negative residual gravity anomaly (Model A2), has an extreme density contrast of 400 kg/m^3^, which is much larger than the expected anomaly (− 200 to − 100 kg/m^3^) from the estimated Vp anomaly (− 10%). The assumed density contrasts in Model A3, where the channel layer has a thickness of 1 km, are well within the plausible range of density variations expected from the Vp profiles. We concluded that a structural model which considers the presence of a low-density channel layer is the most possible model, as it can explain the observed residual gravity anomaly as well as the Vp model obtained in the region without any significant contradictions.

## Effects of the channel layer on the spatiotemporal slip distribution

**Figure 3:**
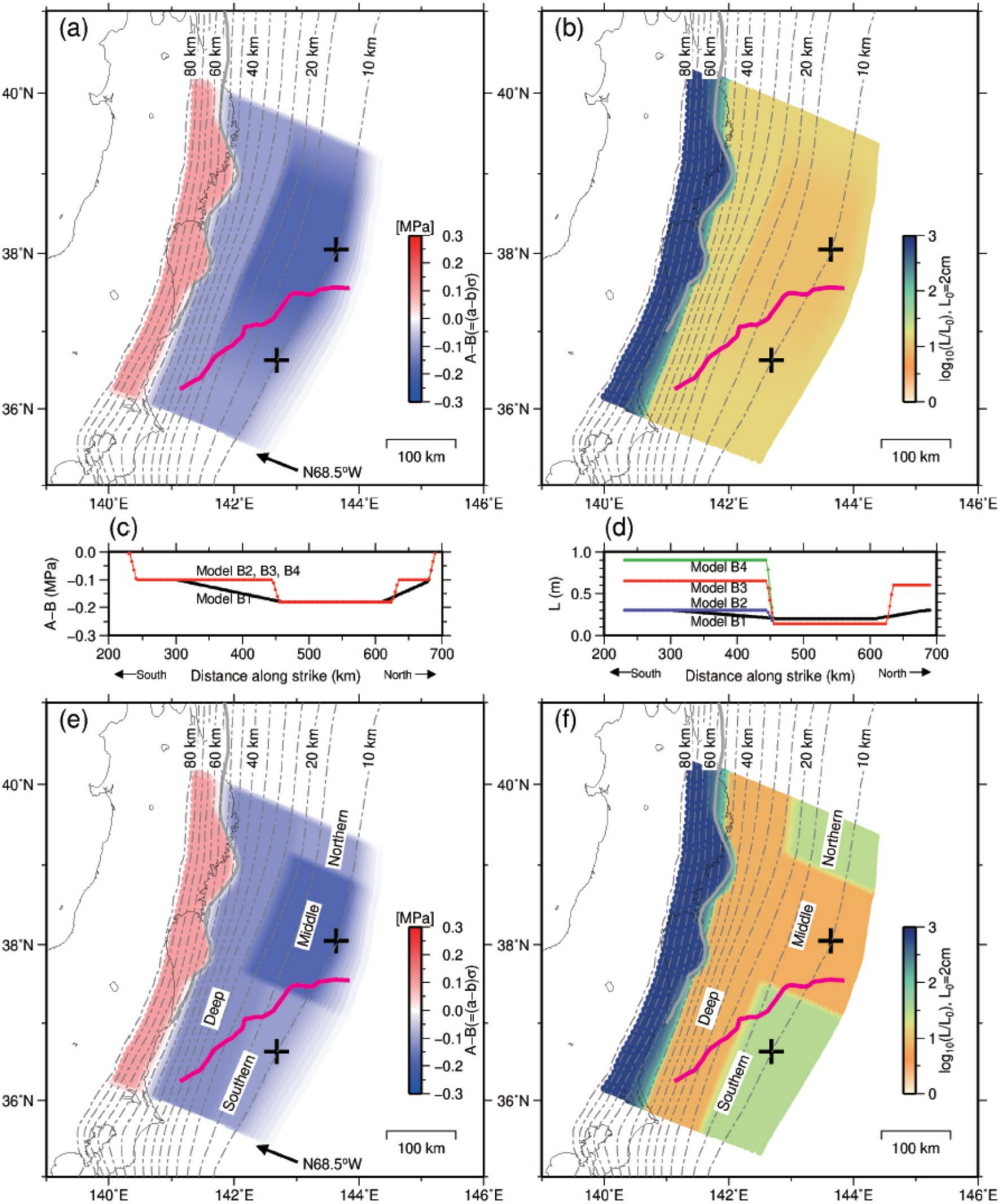
Distribution of frictional parameters for Model B1. (**a**) Spatial distribution on the plate interface of (A–B) (MPa). The magenta lines are the same as in Fig. 1a. Crosses indicate the points shown in Supplementary Fig. 1. Contours indicate the depth (km) to the upper surface of the descending plate^41^. The grey solid line indicates the same as the red line in Fig. 1a. (**b**) Spatial distribution of the characteristic slip distance (L). (**c**)–(**d**) Profiles of A–B and L along the strike at a depth of 10.15 km. Black, purple, red, and green lines represent the Model B1, B2, B3, and B4, respectively. (**e**,**f**) Spatial distribution on the plate interface of (A–B) (MPa) and the characteristic slip distance (L) for Model B3. Crosses indicate the points shown in Fig. 4. The descriptions of segments ‘Northern’, ‘Middle’, ‘Southern’, and ‘Deep’ are the same as those used in Table 1.

**Figure 4:**
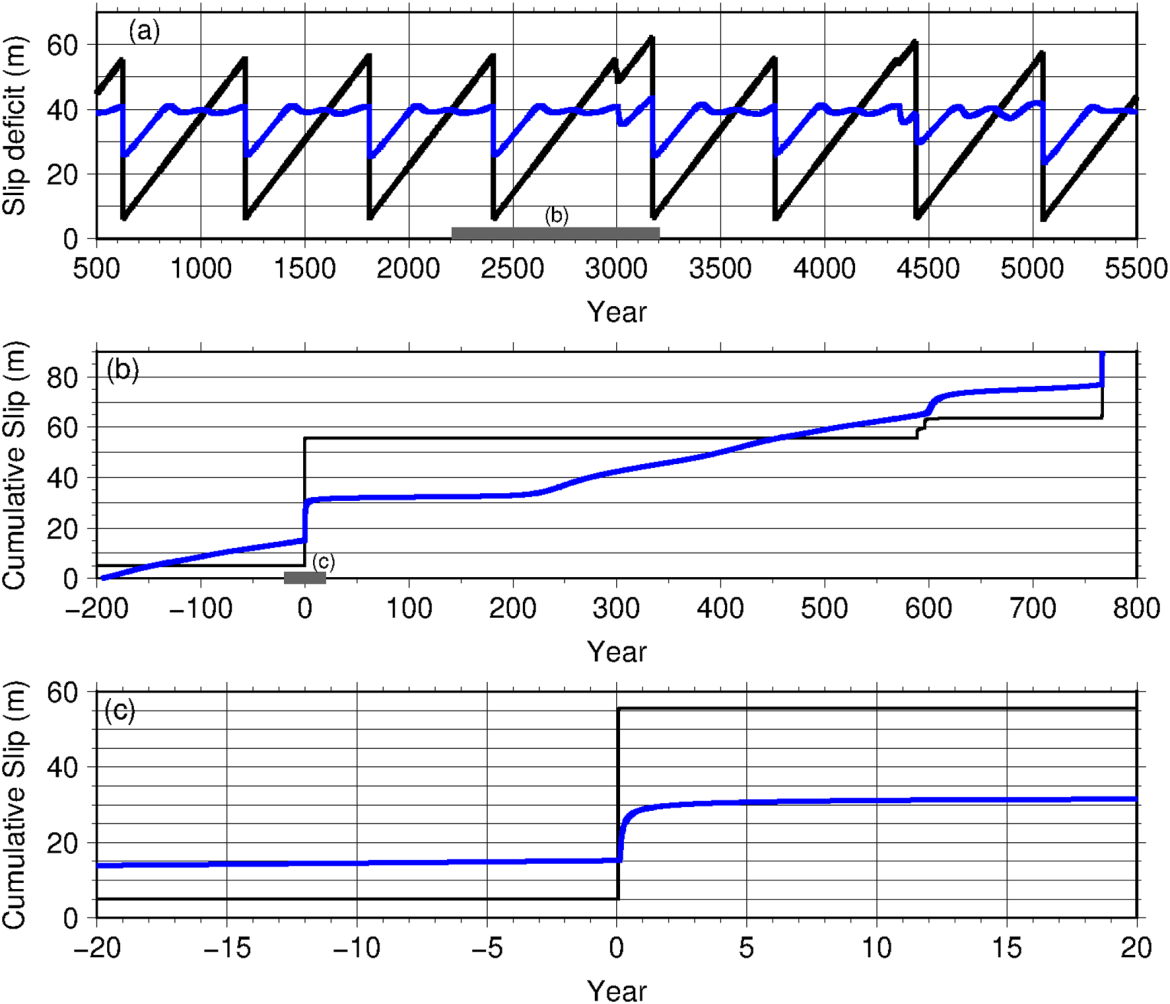
(**a**) Temporal distribution of slip deficits at the point within the M9 source area (black) and southern segment (blue) at the locations of the crosses shown in Fig. 5 over 5000 years for Model B3. Thick, grey line indicates the period shown in (**b**). (**b**,**c**) Temporal variation of the cumulative slip at the point within the M9 source area (black) and southern segment (blue) at the locations of the crosses shown in Fig. 5. (**b**) 200 years before and 800 years after the M9 earthquake obtained for Model B3. Thick, grey line indicates the period shown in (**c**). (**c**) 20 years before and after each M9 earthquake obtained for Model B3.

**Figure 5:**
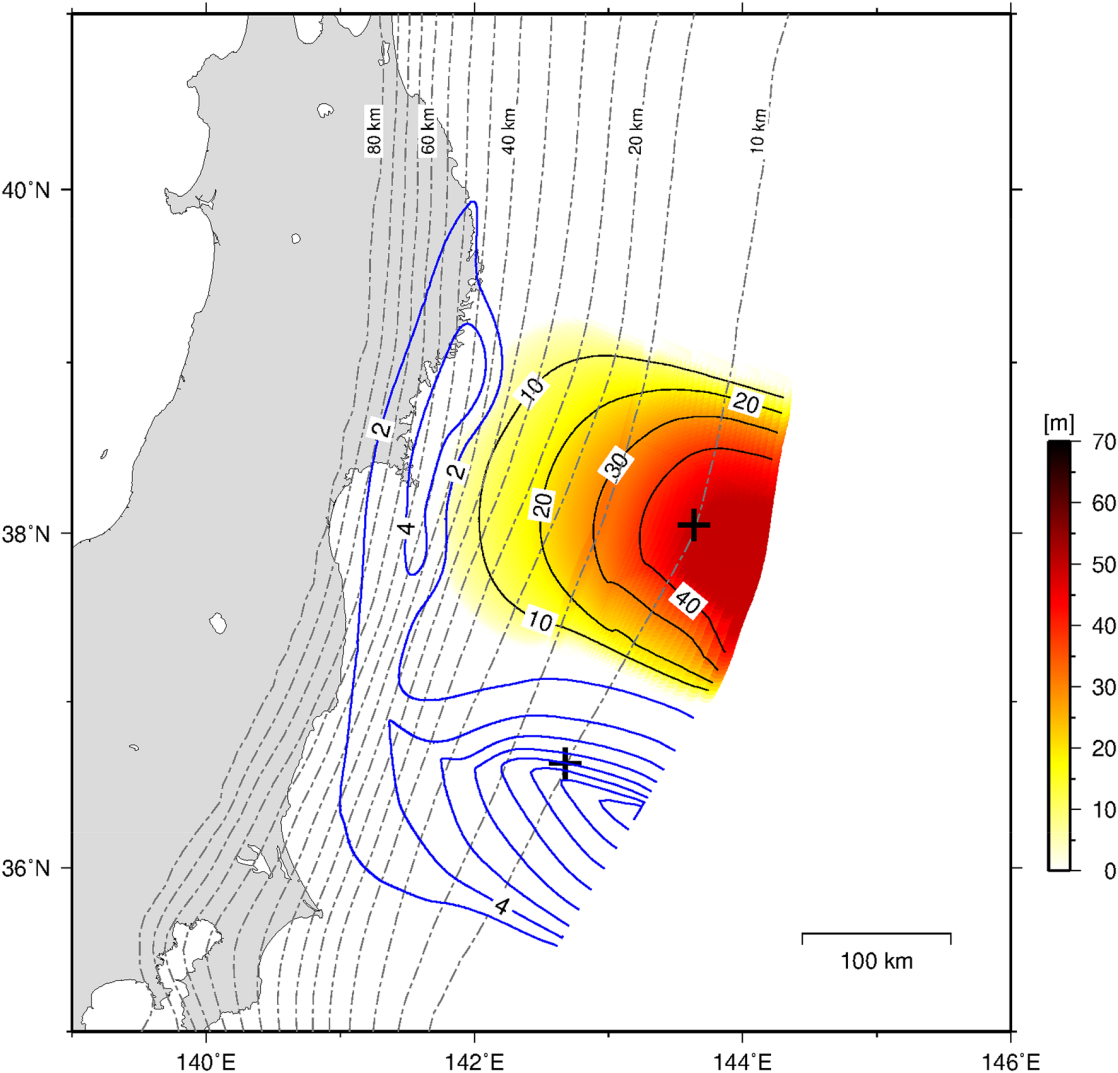
Coseismic slip distribution (when V > 1.0 cm/s) (warm colours) and the postseismic slip (blue contours) of the simulated M9 earthquake for Model B3. Postseismic slips were calculated for 5 years from 0.1 years after each M9 earthquake when Vpl < V < 1.0 cm/s. Crosses indicate the points shown in Fig. 4. The magnitude of each event was 8.91 (T = 2406 year).

We performed calculations using numerical simulations over an extensive period (approximately 5000 years) and identified 7–8 instances of M9 (M = 8.9–9.1) earthquakes, using four models with different parameter settings (Table 1 and Fig. 3). For all the models, the time intervals of the M9 earthquakes were 540–770 years (Fig. 4a,b, and Supplementary Fig. 1a–d, black line). However, coseismic slip areas larger than 10 m were relatively different from a spatial perspective (Fig. 5 and Supplementary Fig. 2). M9 earthquakes in Models B1 and B2 ruptured both middle and southern segments, whereas the rupture areas of Models B3 and B4 were limited to the middle segment of the Japan Trench. The rupture patterns produced by Models B3 and B4 were consistent with the turbidite surveys, which showed depositional events occurring at intervals of 500–900 years over the past 4000 years at the middle segment^6,34^.

**Table 1:** Parameters at the northern, middle, southern, and deep segments for four models.

Segments	Parameters	Model B1	Model B2	Model B3	Model B4
Northern	A–B (MPa)	− 0.10	− 0.10	− 0.10	− 0.10
	b–a	0.0020	0.0020	0.0020	0.0020
	L (m)	0.30	0.60	0.60	0.60
	Rb	0.118	0.118	0.118	0.118
	Ru	0.125	0.271	0.271	0.271
	Length (km) *4	15 (*1)	65 (*3)	65 (*3)	65 (*3)
Middle	A–B (MPa)	− 0.18	− 0.18	− 0.18	− 0.18
	b–a	0.0036	0.0036	0.0036	0.0036
	L (m)	0.20	0.14	0.14	0.14
	Rb	0.194	0.194	0.194	0.194
	Ru	3.389	5.487	5.487	5.487
	Length (km) *4	150	170	170	170
Southern	A–B (MPa)	− 0.10	− 0.10	− 0.10	− 0.10
	b–a	0.0020	0.0020	0.0020	0.0020
	L (m)	0.30	0.30	0.65	0.90
	Rb	0.118	0.118	0.118	0.118
	Ru	0.667	1.875	0.865	0.625
	Length (km) *4	80 (*2)	225 (*3)	225 (*3)	225 (*3)
Deep	A–B (MPa)	− 0.10	− 0.12	− 0.12	− 0.12
	b–a	0.0020	0.0024	0.0024	0.0024
	L (m)	0.30	0.15	0.15	0.15
	Rb	0.118	0.138	0.138	0.138
	Ru	4.0	9.6	9.6	9.6
	Length (km) *4	480	480	480	480

*1: Transition between the middle and northern segments is 75 km.

*2: Transition between the middle and southern segments is 160 km.

*3: Transition between the middle segment and northern/southern segment is 10 km.

*4: Length along strike direction.

Postseismic slip was dominant in the shallow part of the southern segment in the results of Models B3 (Fig. 5) and B4 (Supplementary Fig. 2c) and continued for more than 5 years in both Model B3 (Fig. 4c, blue line, and Supplementary Fig. 3) and Model B4 (Supplementary Fig. 1e, green line, and Supplementary Fig. 4). The long duration of postseismic slip is consistent with geodetic observation^13^. The reproducibility of these results is due to the large contrast in frictional parameters between the middle and southern segments [the larger value of the characteristic slip distance, L, and a steeper change in frictional parameters (Model B3; Table 1, and Fig. 3e,f)].

Considering a gradual variation in L between the middle and southern segments (Model B1; Table 1 and Fig. 3), the M9 coseismic slip propagated southward, and postseismic slip did not dominate at the southern segment (Supplementary Fig. 2a). When we adopted the segmentation boundary with a smaller contrast of frictional parameters (Model B2 in Table 1 and Fig. 3d, purple line), the resulting slip distribution was similar to that of Model B1 (Supplementary Fig. 2b), and postseismic slip at the southern segment quickly terminated (Supplementary Fig. 1e, purple line and 5).

However, the extremely large contrast in L between the two segments is not consistent with the postseismic slip observations. When we used a greater value of L in the southern segment (Model B4 in Table 1, and Fig. 3d, green line), M9 coseismic slip was limited to the middle segment, which is similar to Model B3 (Supplementary Fig. 2c). However, the propagation of postseismic slip to the south would take a longer time than in Model B3 (Supplementary Fig. 4). Consequently, the postseismic slip initiated in the southern segment approximately 0.5 years after the simulated M9 earthquake (Supplementary Fig. 1e, green line). The significantly delayed onset of postseismic slip is not consistent with the observation of the 2011 Tohoku-Oki earthquake estimated from small repeating earthquake activity, which increased shortly after the mainshock^35^ although the exact timing of the onset is not evident from geodetic observations. Therefore, the temporal distribution of postseismic slip could constrain the contrast of L between the middle and southern segments.

When the contrast of L between the two segments was similar to that in Model B3, we could explain the significant difference in the coseismic and postseismic slip distributions along the trench and the long duration of the postseismic slip, as observed. The spatiotemporal distributions of coseismic and postseismic slips of Model B3 were roughly consistent with geodetic observations^3,8^. However, in our simple model, the amount of postseismic slip was considerably larger than that observed in the southern segment^7,12^. However, there are large uncertainties in both observations and simulations. Although the present modelling indicated that most of the slippage occurred immediately after the M9 earthquake, it is very difficult to constrain aseismic fault motion outside the coseismic rupture area in the very early postseismic period from seismic and geodetic observations. Early postseismic behaviour may be subject to the treatments of dynamic rupture and/or viscoelasticity of the earth. For example, dynamic weakening processes, such as thermal pressurisation^36,37^ and viscoelastic relaxation^12,38,39^, have to be carefully considered to quantify coseismic/postseismic slips in detail. In addition, in our numerical simulation modelling of the earthquake generation cycle under the condition of velocity weakening, we assumed that only L, and not both |A–B| and L, would be more attributed to the channel layer. Thus, our model may be over-simplified to represent postseismic slips. It may be necessary to include these physical conditions and small-scale structural heterogeneities in our future model.

## Conclusions

In this study, we demonstrated that the presence of a thick, low-velocity channel layer in the subducting plate at the southern segment of the Japan Trench is consistent with gravity anomaly distribution characteristics, which accounts for the significantly different slip behaviour of the middle and southern segments near the trench axis. A structural model that considers the presence of the channel layer is the best way to explain the observed residual gravity anomaly and seismic velocity without any significant contradictions. By representing the presence of the channel layer as the value of a characteristic slip distance in the rate- and state-dependent frictional law, we efficiently reproduced the longer duration of the postseismic slip of the 2011 Tohoku-Oki earthquake, as well as the spatial distributions of the complementary coseismic and postseismic slips at the middle and southern segments, maintained for recurrence intervals of ~ 600 years.

## Method of estimation of density distribution

In the present study, we sought to create 2D density anomaly models which explain the difference in the residual gravity anomaly across the forearc segment boundary (FSB) presented by Bassett et al.^15^ in the southern segment of the Japan Trench, where postseismic slip is evident. We calculated gravity anomalies using three different density distribution models (Models A1, A2, and A3, in Fig. 2a) using the classical Talwani method^40^ and compared them to the observed residual gravity anomaly. The crustal layer is assumed to have a thickness of 10 km because the depth to the plate boundary is ~ 10 km below the seafloor in the postseismic slip zone. We included the observed gravity anomaly in the region with a plate boundary depth which ranged from 9 to 11 km. We created a 2D profile along the trench axis, and set *x* = 0 at the location of the FSB. A negative density anomaly was considered for the southern half of the models (*x* < 0). Model A1, which is the simplest model, had a homogeneous density anomaly of 180 kg/m^3^ in the crust and was similar to the model used by Bassett et al.^15^ to interpret the observed gravity anomaly. Model A2 had a thinner layer of density anomaly, emulating the lower sedimentary layer imaged as the low-Vp layer delineated by seismic explorations (Fig. 1c). Model A3 had two layers of negative density anomaly: the first was the sedimentary layer corresponding to that in Model A2, but with a smaller anomaly, and the second was below the base of the crustal layer. The calculated gravity anomaly was adjusted by adding 40 mGal (Fig. 2b) because we were interested in the degree of the difference in the residual gravity anomaly across the FSB, and not in the absolute values of the anomaly.

## Method of simulating earthquake generation cycles

We conducted numerical simulations of earthquake generation cycles using the realistic three-dimensional (3D) geometry of the subducting Pacific Plate along the Japan Trench^41^. Detail of simulating methods were given in Supplementary text, we used the same equations, initial conditions, seismic radiation damping term, plate geometry, and plate convergence rate as in our previous study^42^, which roughly approximated several characteristics of M7 earthquakes (such as the 1978 Miyagi–Oki earthquake) and M9 earthquakes (such as the 2011 Tohoku-Oki earthquake) in the middle segment of the Japan Trench. Seismic and aseismic events were modelled to represent the release of the slip deficit or backslip which accumulates during interseismic periods^43^.

Such spatiotemporal variations in the slip velocity were assumed to indicate an unstable slip with a frictional interface. We used a rate- and state-dependent frictional law^27^ as an approximate mathematical model for large-scale frictional behaviour at the plate interface and a fault constitutive law^44^ to determine the slip rate for a given stress and strength value. In addition, we used an aging law^27,45^, which can be considered an evolution law for changes in strength that varies depending on prior slip history.

In our simulations of earthquake generation cycles, we used the quasi-dynamic (QD) model^43^ with a smaller radiation damping term instead of a fully dynamic (FD) model. The QD model typically cannot simulate fast slips during seismic events; however, in models with standard rate-and-state friction and relatively uniform fault properties, the FD and QD simulations produce qualitatively similar fault behaviours, with crack-like ruptures and similar earthquake patterns^46^.

In many models, afterslip is often assumed to occur in the regions of velocity-strengthening frictional property^37,47^. Although slow slip events (SSEs) can also occur under the conditions of velocity weakening and large nucleation size^48–50^, velocity strengthening with a one-state variable in the rate- and state-dependent law cannot cause SSE. We did not rule out the possibility of slow slips (not postseismic slip) occurring in the shallow area of the southern segment during the M9-interseismic period. Then, similar to our previous studies^28,29,42,51^, we adopted the frictional condition of velocity weakening in the afterslip area. Small-scale heterogeneity must be introduced to generate small slow earthquakes (low-frequency earthquakes, tectonic tremors, and very-low-frequency earthquakes) in both models (velocity strengthening and weakening).

During our simulations, we only changed the numerical values and the spatial distributions of the frictional parameters (A, B, and L), focussing on the slip in the shallow southern area. The parameter A (= aσ) controls the slip increase rate at which the stress reaches the strength. We did not directly consider the effective normal stress σ because its effect is difficult to separate from that of the frictional parameter a (and b, as will be described below) for natural earthquakes. Parameters B (= bσ) and L control strength recovery and slip weakening. Nakata et al.^42^ approximated the spatial distributions of frictional parameters for seismic sources with circular patches for M7 earthquakes, which repeatedly occurred at intervals of several decades in the past. For comparison, in the present study, we removed these circular patches for simplicity and used the model without these unstable patches (Model B1, Fig. 3a,b, Table 1), instead of the one reported by Nakata et al.^42^. The calculation time was then reduced so that we could calculate multiple cycles of the M9 earthquakes. In a method similar to that reported by Nakata et al.^42^, the M9 coseismic rupture area located in the middle segment of the Japan Trench was approximated by a rectangle (an area 150-km long along the strike and shallower than 22 km in depth). At the boundary between the southern and northern sides of the M9 source area, the frictional characteristics gradually changed, and differences between the deep and shallow areas were not considered.

For Model B3 (Fig. 3e,f, Table 1), the values and spatial distribution of the frictional parameters (A, B, and L) were determined with reference to the observations of the residual gravity anomaly by Bassett et al.^15^. The M9 coseismic rupture area was approximated by a rectangle (170 km along the strike and a depth shallower than 24 km). Here, we divided the surrounding area, which we assumed to have a uniform frictional condition as in Model B1, into three segments: northern, southern, and deep segments (Fig. 3e,f). The difference in frictional parameters at the boundaries between the northern, southern, and middle (the M9 coseismic) segments were then assumed to be larger than those of Model B1 (Fig. 3c,d), and their locations were based on an observed residual gravity anomaly which is less than zero. Spatial distributions of the coseismic slip, postseismic slip, seismic velocity, and residual gravity anomaly showed that uniform and smooth assumptions of frictional boundaries may not be appropriate. Boundary settings along the trench between the middle and southern segments were the main differences between Model B1 and Model B3. In addition, based on our hypothesis, the frictional parameter controlling slip weakening (L) at the southern segment is assumed to be larger than that of the middle and deep segments. By employing iterative processes of trial and error, fine-tuning of the frictional parameters was undertaken between Models B1 and B3.

To assess the effect of the channel layer, we conducted calculations using four models (Table 1). Model B1 was almost the same as in our previous study. This model did not have a channel layer. Models B2, B3, and B4 were the modified models used in this study. Model B2 had a small L value (the same as Model B1), Model B3 had a medium L value, and Model B4 had a large L value in the southern segment. Model B2 had a channel layer in which the contrast between the middle and southern segments was weak. Models B3 and B4 represented models with a high-contrast channel layer. The L values in the southern segment were 2.1, 4.6, and 6.4 times larger than in the middle segment for Models B2, B3, and B4, respectively. Although both L and |A–B| affect slip behaviour, the frictional parameter which appears to be related to the thick channel layer (surface roughness) is considered to be L instead of |A–B|. Therefore, in this study, we exclusively compared the effect of L.

## Supplementary information

Supplementary information.

## Data Availability

The datasets generated during and/or analysed during the current study are available from the corresponding author on reasonable request.
