# Supplementary material for: Presence of interplate channel layer controls of slip during and after the 2011 Tohoku-Oki earthquake through the frictional characteristics
Source: Sci Rep. 2021 Mar 19;11:6480. doi: 10.1038/s41598-021-86020-9 (PMC7979718; doi:10.1038/s41598-021-86020-9)
Supplement: Supplementary file 1 — Supplementary information. [file 41598_2021_86020_MOESM1_ESM.pdf]

Supplementary information

Presence of interplate channel layer controls of slip during and after the 2011 Tohoku-Oki earthquake through the frictional characteristics

Ryoko Nakata[1*], Takane Hori[2], Seiichi Miura[3], and Ryota Hino[1]

[1] Graduate School of Science, Tohoku University, 6-6, Aramaki-aza-aoba, Aoba-ku, Sendai 980-8578, Japan

*e-mail: ryoko.nakata.e6@tohoku.ac.jp

[2] Research and Development Center for Earthquake and Tsunami Forecasting (FEAT), Research Institute for Marine Geodynamics (IMG), Japan Agency for Marine-Earth Science and Technology (JAMSTEC), 3173-25 Showa-machi, Kanazawa-ku, Yokohama 236-0001, Japan

[3] Subduction Dynamics Research Center (SDR), Research Institute for Marine Geodynamics (IMG), Japan Agency for Marine-Earth Science and Technology (JAMSTEC), 3173-25 Showa-machi, Kanazawa-ku, Yokohama 236-0001, Japan

Supplementary Figures and Figure legends

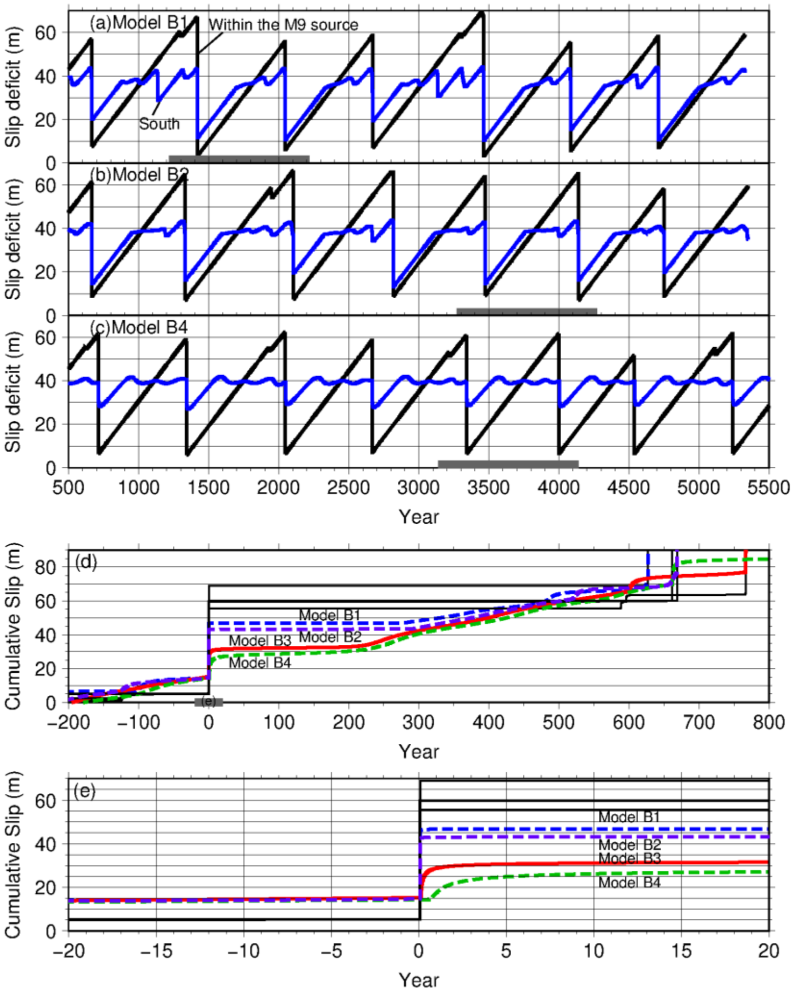

Supplementary Figure 1. (a)–(c) Temporal distribution of slip deficits at the point within the M9 source area (black) and southern segment (blue) over 5000 years for Models B1, B2, and B4, respectively. Thick, grey lines indicate the periods shown in (d). (d)–(e) Temporal variation of the cumulative slip at the crosses shown in Figs. 3 and 5, and Supplementary Fig. 2, the point within the M9 source area (black lines) and southern

segment (colored lines). For comparison, the cumulative slips for Model B3 are also shown by the red lines, which are the same as the blue lines in Fig. 4b–c. (d) 200 years before and 800 years after the M9 earthquake. (e) 20 years before and after each M9 earthquake obtained for Model B1 (blue), B2 (purple), B3 (red), and B4 (green). These temporal characteristics at the southern segment (recurrence interval of M9 earthquake and perturbation at the later stage of a cycle) were almost the same among four models.

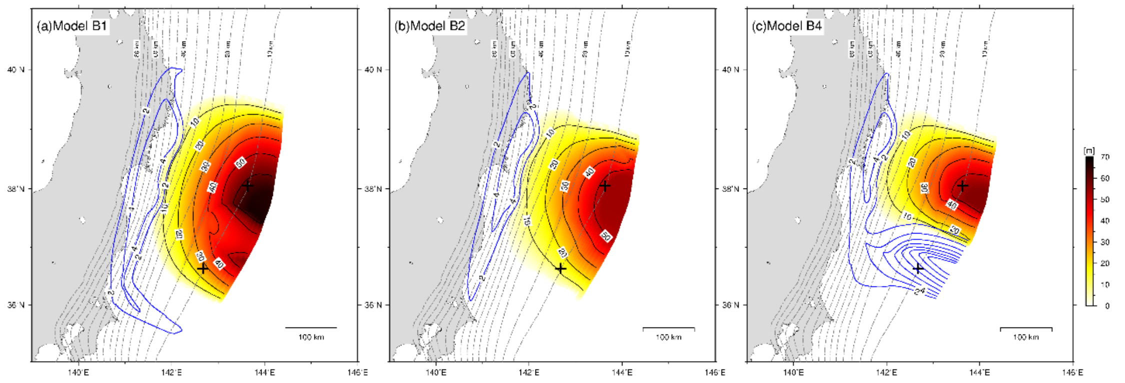

Supplementary Figure 2. Coseismic slip (when $V > 1.0$ cm/s) distribution (warm colors) and postseismic slip (blue contours) of the simulated M9 earthquake for (a)–(c) Models B1, B2, and B4, respectively. Postseismic slips were calculated for 5 years from 0.1 years after each M9 earthquake when $Vpl < V < 1.0$ cm/s. Crosses indicate the points shown in Supplementary Fig. 1. The magnitude of each event was 9.13 (T = 1418 yr), 9.03 (T = 3471 yr), and 8.92 (T = 3340 yr) for Models B1, B2, and B4, respectively. There was no postseismic slip at the southern segment in Model B2 because postseismic slip did not occur during the period used for drawing (postseismic slip almost converged within 0.1 years after the M9 earthquake). On the other hand, there was no postseismic slip at the shallow southernmost part of Model B4 because the postseismic slip had not yet propagated to that location.

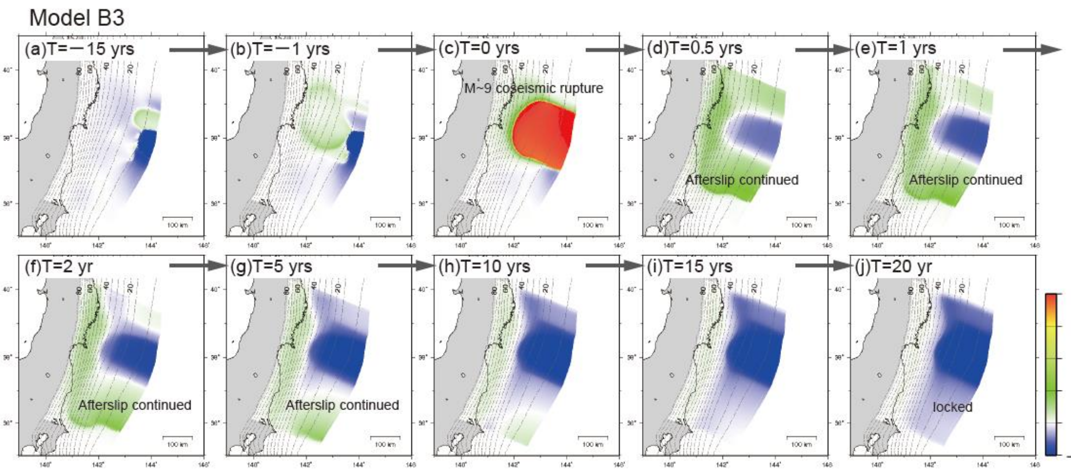

Supplementary Figure 3. Slip velocity distribution prior to, during, and following the M9 earthquake obtained by numerical simulation using Model B3. (a)–(b) 15 years and 1 year prior to the M9 earthquake. (c) Coseismic rupture of the M9 earthquake propagated. (d)–(j) 0.5, 1, 2, 5, 10, 15, and 20 years after the M9 earthquake, respectively. The blue and red areas indicate the locked parts of the fault and unstably slipping parts of the fault, respectively. Yellow/green and white indicate slow slip and plate convergence rates, respectively.

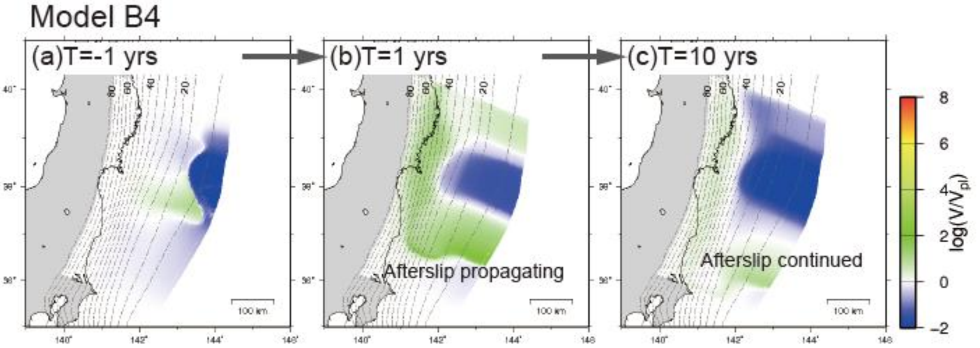

Supplementary Figure 4. Slip velocity distribution prior to and following the M9 earthquake obtained by numerical simulation using Model B4. (a) 1 year prior to the M9 earthquake. (b)–(c) 1 and 10 years after the M9 earthquake.

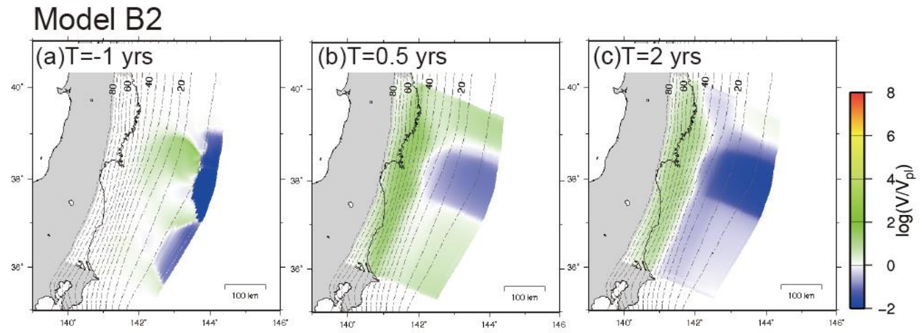

Supplementary Figure 5. Slip velocity distribution prior to and following the M9 earthquake obtained by numerical simulation using Model B2. (a) 1 year prior to the M9 earthquake. (b)–(c) 0.5 and 2 years after the M9 earthquake.

Supplementary Text 1

1.1. Equations and parameters used in simulating earthquake generation cycles

We discretised the subducting plate into small subfaults in which the length in a given direction (N21.5°E) varied with depth or strike, ranging from 1.0 km to 9.0 km (Supplementary Text 1.2). Hereafter, subscripts *i* and *j* denote the subfaults modelled for the simulation. The time derivative of shear stress τ on the *i*-th subfault owing to the slip at the *j*-th subfault is calculated as follows:

$$\frac{d\tau_i}{dt} = \sum_j K_{ij}\left(V_j - V_{pl,j}\right) - \eta\frac{dV_i}{dt}, \qquad (1)$$

where K_{ij} is the stress increment on a subfault *i* owing to unit slip on a subfault *j*, V_j is the slip rate of the *j*-th subfault in the plate convergence direction, $V_{pl,j}$ is the plate convergence rate of the *j*-th subfault, and η denotes the seismic radiation damping term [Rice, 1993]. We used $\eta = 0.3G/2\beta$, G is the rigidity (30 GPa), and β is the shear wave speed (3.27 km/s). The Poisson's ratio is 0.25. We set the plate convergence rate as V_{pl} = 8.4 cm/yr [Ohtani et al., 2014].

Such spatiotemporal variations in the slip velocity were assumed to indicate an unstable slip with a frictional interface. We used a rate- and state-dependent frictional law [Dieterich, 1979] as an approximate mathematical model for large-scale frictional

behaviour at the plate interface.

$$V_i = V^* \exp\left[\frac{\tau_i - (\tau_{s*i} + \Delta\tau_{si})}{A_i}\right], \tag{2}$$

$$\frac{d\Delta\tau_{si}}{dt} = \frac{B_i}{L_i/V^*} \exp\left(-\frac{\Delta\tau_{si}}{B_i}\right) - B_i \frac{V_i}{L_i}. \tag{3}$$

Equation (2) represents a fault constitutive law [Nakatani, 2001] which determines the slip rate V_i for a given stress τ_i and a value of τ_{si} (= $\tau_{s*i} + \Delta\tau_{si}$). When we set the reference velocity V^* at a slip velocity level below at which the slip is sufficiently slow to be negligible, the value of τ_{si} is the threshold level of the stress τ_i required to cause significant slip velocity. Therefore, the value of τ_{si} is analogous to the 'strength as a threshold' [Nakatani, 2001], and we refer to it simply as 'strength'. The parameter A (= $a\sigma$) controls the slip increase rate at which the stress reaches the strength. We did not directly consider the effective normal stress σ because it is difficult to differentiate its effect from that of the frictional parameter a (and b, as will be described below) for natural earthquakes. V^* was set to V_{pl} in the calculation below; here, τ_{s*} (= $\mu^*\sigma$) represents the steady-state strength with $V = V^*$, and $\Delta\tau_s$ is the variation in strength from the steady state.

Equation (3) is an ageing law [Dieterich, 1979; Ruina, 1983]. When strength is defined

in this manner, equation (3) can be considered as an evolution law for strength change $\Delta\tau_s$, which varies depending on the prior slip history. Parameters B (= $b\sigma$) and L control strength recovery and slip weakening. For slip weakening, B and L primarily determine the amplitude of strength variation $\Delta\tau_s$ and slip weakening distance D_c. These are the minimum characteristics of large-scale friction and are caused by various small-scale physical processes. From this perspective, frictional parameters A (= $a\sigma$), B (= $b\sigma$), and L in the rate- and state-dependent friction law are mathematical fitting parameters, which were assumed to be constant over the earthquake cycle in our simulations.

To solve equations (1)–(3), we removed the time derivative of stress $d\tau_i/dt$ and obtained differential equations for slip rate V and strength $\Delta\tau_s$. Assuming the initial values given below, differential equations were solved with an adaptive time step fifth-order Runge–Kutta algorithm [Press et al., 1996]. For the initial conditions, the slip velocity V was assumed to be uniform at $0.9V_{pl}$, and the strength was calculated as $\tau_{s*i} + \Delta\tau_{si} = \Delta\tau_{si} = -B_i\ln(V_i/V_{pl})$. For the boundary conditions, the slip velocity was assumed to be constant and equal to V_{pl} outside the model region.

1.2. Geometry of the plate interface

We used a discretized 3D Pacific plate interface geometry [Baba et al., 2006] to

calculate the slip response function Kij. The plate interface was first divided into small rectangular subfaults, each of which was approximated by three triangles to allow calculation of angular dislocation [Comninou and Dundurs, 1975]. Slip response functions are represented by the stress change on a subfault, which is represented here by the combined effect of three angular dislocations within a subfault. This stress change was estimated at the centre of each subfault in the direction of slip acceleration. The slip responses Kij were evaluated in a homogeneous elastic half space.

The model presented here is from Iwate Prefecture to Ibaraki Prefecture. The length of the domain in the strike direction (N21.5°E) was approximately 480 km on the shallower side, and the depth range of the model region was 7–77 km (Figure 3 in the main text). Small subfaults were set with lengths of 1.0 km and 0.1 km in the strike and depth directions (i.e., vertical to the horizontal plane), respectively. The edge of the modelled region was set parallel to the direction of the subducting plate (N68.5°W) (arrows in Figures 3a and e). The total number of subfaults equalled 336,000; however, we reduced this number to shorten the calculation time by adopting large sizes for apparent subfaults. Of the 480 km length of the strike direction, 360 km on the south side of the model region, we then calculated the stress changes by summing the effects of nine (3 × 3) small subfaults (length of each apparent subfault in the strike direction is 3.0 km) at depths of

7–13 and 44–59 km, and 81 (9 × 9) small subfaults (length in the strike direction is 9.0 km) at depths of 59–77 km. For the remaining 120 km on the north side of the model, we used subfaults that combine the nine and 81 smallest subfaults at depths of 7–59 km and 59–77 km, respectively. Consequently, the total number of apparent subfaults was reduced to 127,367.

1.3. Frictional property distribution

The frictional property distribution is set here with reference to Nakata et al. [2016]. We assumed depth-dependent heterogeneity of $A–B$ (A and B are defined in Section 1.1). As shown in Figure 3, $A–B < 0$ (i.e. velocity weakening in steady-state slip) was set for depths of 7–46 km of the model region. We set $A–B > 0$ for depths > 62 km. Between 46 and 62 km depth, $A–B$ gradually changed from negative to positive. Subsequently, this area was adjusted with reference to the western limit of the interplate earthquake distribution by Igarashi et al. [2001] (the grey solid line in Figures 3a, b, e, and f). We set L = 13 m in the $A–B > 0$ region. Areas with a large L (13 m) satisfy the conditions of stable sliding.

In the shallowest boundary of the model region, we set $A–B = 0$. Both boundaries of the model along the strike direction were also $A–B = 0$ (10 km inward from the model

boundary). *A–B* gradually transitions from 0 to an arbitrary value between 10 km and 20 km inward from the model boundary. *L* remained constant at the boundary.

The characteristics of the simulated slip were controlled by the nondimensional numbers *Ru* and *Rb*, which are expressed by equations (4) and (5) [Barbot, 2020].

$$\mathrm{R_b} = \frac{(b-a)}{b} \qquad (4)$$

$$\mathrm{R_u} = \frac{(b-a)\sigma}{\mathrm{G}}\frac{\mathrm{W}}{\mathrm{L}}(1-\nu) \qquad (5)$$

a, *b*, *L*, and *G* are the same as in Section 1.1. Although *W* is the width of the velocity-weakening patch, it is treated here as the length along the strike direction. ν is the Poisson's ratio (0.25), and σ is the effective normal stress accounting for the effect of pore pressure (50 MPa at depths greater than 10 km). When *Ru* >> 1, the fault slip is unstable. Conversely, it is quasi-stable when *Ru* is close to 1 and stable when *Ru* << 1 [Barbot, 2020]. A low positive *Rb* number is associated with conditions approaching a neutral velocity [Barbot, 2020]. A large *Rb* number approaching 1 from below corresponds to strong weakening unless *a* is abnormally large [Barbot, 2020]. We assumed heterogeneity of *A–B* and/or *L* for the northern, middle, southern, and deep segments to satisfy the above

conditions for *Rb* and *Ru*. In addition to *A*–*B* and *L*, these nondimensional parameters are also shown in Table 1.

The length and width of the M9 seismogenic zone were defined based on various studies [e.g., Ide et al., 2011; Suito et al., 2011; Suzuki et al., 2011; Yagi & Fukahata, 2011; Iinuma et al., 2012; Ozawa et al., 2012; Yamagiwa et al., 2015; Wang et al., 2016]. Consequently, for Models B2–B4, we set a depth of 20 km as the deeper limit of the seismogenic zone. The length of the seismogenic zone was approximately 170 km along the strike. We set *L* = 0.14 m, *A*–*B* = –0.18 MPa in this zone. The values of *Ru* and *Rb* at a point in the source region of the Tohoku earthquake (cross in Figure 2a) were approximately 5.487 and 0.194, respectively, such that *Ru* >> 1 and low *Rb*. M9 earthquakes were then expected to occur within this unstable zone.

For the southern segment of Models B2–B4, we assumed a depth of 15 km as the deeper limit. The horizontal length was 225 km in the strike direction. In the southern segment, we assumed a quasi-stable condition, *L* = 0.30–0.90 m, *A*–*B* = –0.10 MPa, *Ru* = 0.6–1.9 (close to 1), *Rb* = 0.118. Thus, aseismic slips are expected to occur in the southern segment. For the northern segment of Models B2–B4, we assumed a depth of 20 km as the deeper limit. The horizontal length was 65 km in the strike direction. In the northern segment, *L* = 0.60 m, *A*–*B* = –0.10 MPa, *Ru* = 0.271 (*Ru* << 1), *Rb* = 0.118. In this study,

we focused on the middle and southern segments, and then we set stable conditions in the northern segment. In the deep segment of Model B2–B4, where many M > 7 earthquakes occurred in the past, L = 0.15 m, A–B = –0.12 MPa, Ru = 9.6 (Ru >> 1), Rb = 0.138.

References

Rice, J. R. Spatio-temporal complexity of slip on a fault. *J. Geophys. Res*. **98,** 9885–9907 (1993).

Ohtani M., Hirahara, K., Hori, T. & Hyodo, M. Observed change in plate coupling close to the rupture initiation area before the occurrence of the 2011 Tohoku earthquake: Implications from an earthquake cycle model. *Geophys*. *Res*. *Lett*. **41**, 1899-1906 (2014).

Dieterich, J. H. Modeling of rock friction, 1. Experimental results and constitutive equations. *J. Geophys. Res.* **84,** B5, 2161–2168 (1979).

Nakatani, M. Conceptual and physical clarification of rate and state friction: Frictional sliding as a thermally activated rheology. *J. Geophys. Res*. **106,** 13347–13380 (2001).

Ruina, A. Slip instability and state variable friction laws. *J. Geophys. Res*. **88,** 10359–10370 (1983).

Press, W. H., Teukolsky, S. A., Vetterling, W. T. & Flannery, B. P. in *Numerical Recipes*

in Fortran 77: The Art of Scientific Computing (Vol. 1 of Fortran Numerical Recipes). Cambridge University Press (1996).

Baba, T., Ito, A., Kaneda, Y., Hayakawa, T. & Furumura, T. 3-D seismic wave velocity structures in the Nankai and Japan Trench subduction zones derived from marine seismic surveys. *Japan Geoscience Union Meeting* S111–006 (2006).

Comninou, M. & Dundurs, J. The angular dislocation in a half space. *J. Elasticity* **5**, 3-4, 203–216 (1975).

Ide, S., Baltay, A. & Beroza, G. C. Shallow dynamic overshoot and energetic deep rupture in the 2011 Mw 9.0 Tohoku-Oki earthquake. *Science* **332**, 1426 (2011).

Suito, H., Nishimura, T., Tobita, M., Imakiire, T. & Ozawa, S. Interplate fault slip along the Japan Trench before the occurrence of the 2011 off the Pacific coast of Tohoku Earthquake as inferred from GPS data. *Earth Planets Space* **63**, 615-619 (2011).

Suzuki, W., Aoi, S., Sekiguchi, H. & Kunugi, T. Rupture process of the 2011 Tohoku-Oki mega-thrust earthquake (M9.0) inverted from strong-motion data. *Geophys. Res. Lett.* **38**, L00G16 (2011).

Yagi, Y. & Fukahata, Y. Rupture process of the 2011 Tohoku-oki earthquake and absolute elastic strain release. *Geophys. Res. Lett.* **38**, L19307 (2011).

Iinuma, T. et al. Coseismic slip distribution of the 2011 off the Pacific Coast of Tohoku

Earthquake (M9.0) refined by means of seafloor geodetic data. *J. Geophys. Res.* **117**, B07409 (2012).

Ozawa, S. et al. Preceding, coseismic, and postseismic slips of the 2011 Tohoku earthquake, Japan. *J. Geophys. Res.* **117**, B07404 (2012).

Yamagiwa, S., Miyazaki, S., Hirahara, K. & Fukahata, Y. Afterslip and viscoelastic relaxation following the 2011 Tohoku-oki earthquake (Mw9.0) inferred from inland GPS and seafloor GPS/Acoustic data. *Geophys. Res. Lett.* **42**, 66–73 (2015).

Wang, Z., Kato, T., Zhou, X. & Fukuda, J. Source process with heterogeneous rupture velocity for the 2011 Tohoku-Oki earthquake based on 1-Hz GPS data. *Earth Planets and Space* **68**, 193 (2016).
